# Mesangiogenic progenitor cells are forced toward the angiogenic fate, in multiple myeloma

**DOI:** 10.18632/oncotarget.27285

**Published:** 2019-11-26

**Authors:** Simone Pacini, Marina Montali, Francesco Mazziotta, Claudia P. Schifone, Lucia Macchia, Vittoria Carnicelli, Francesca M. Panvini, Serena Barachini, Laura Notarfranchi, Giovanni Battista Previti, Gabriele Buda, Mario Petrini

**Affiliations:** ^1^Department of Clinical and Experimental Medicine, Hematology Division, University of Pisa, Pisa, Italy; ^2^GeNOMEC School of Doctorate, University of Siena, Siena, Italy; ^3^Department of Laboratory Medicine, Azienda Ospedaliero-Universitaria Pisana, Pisa, Italy; ^4^Department of Surgical, Medical, and Molecular Pathology and Critical Care Medicine, University of Pisa, Pisa, Italy; ^5^Institute of Life Sciences, Sant’Anna School of Advanced Studies, Pisa, Italy; ^6^Department of Medicine and Surgery, Hematology Division, University of Parma, Parma, Italy; ^7^Unit of Anesthesia and Intensive Care, University of Sassari, Sassari, Italy

**Keywords:** multiple myeloma, bone marrow microenvironment, angiogenesis, osteogenesis, mesangiogenic progenitor cells

## Abstract

Multiple myeloma (MM) progresses mainly in the bone marrow where the involvement of a specific microenvironment plays a critical role in maintaining plasma cell growth, spread, and survival. In active disease, the switch from a pre-vascular/non-active phase to a vascular phase is coupled with the impairment of bone turnover. Previously, we have isolated *Mesangiogenic Progenitor Cells* (MPCs), a bone marrow population that showed mesengenic and angiogenic potential, both *in vitro* and *in vivo*. MPC differentiation into musculoskeletal tissue and their ability of sprouting angiogenesis are mutually exclusive, suggesting a role in the imbalancing of the microenvironment in multiple myeloma.

MPCs from 32 bone marrow samples of multiple myeloma and 23 non-hematological patients were compared in terms of frequency, phenotype, mesengenic/angiogenic potential, and gene expression profile. Defective osteogenesis was recorded for MM-derived MPCs that showed longer angiogenic sprouting distances respect to non-hematological MPCs, retaining this capability after mesengenic induction. This altered MPCs differentiation potential was not detected in asymptomatic myelomatous disease.

These *in vitro* experiments are suggestive of a forced angiogenic fate in MPCs isolated from MM patients, which also showed increased sprouting activity. Taking together our results suggest a possible role of these cells in the “angiogenic switch” in the MM micro-environment.

## INTRODUCTION

Tumor microenvironment contributes to disease progression in most haematological malignancies [1]. In particular, in Multiple Myeloma (MM) the interactions between malignant plasma cells (PCs) and the bone marrow (BM) niche sustain and promote tumor growth [2]. Endothelial cells, stromal cells, osteoblasts, osteoclasts, and immune cells together with the extracellular matrix are involved in the process. Cross-talks between neoplastic cells and BM create a suitable microenvironment for disease development and are responsible for the hallmarks of MM progression: osteolysis and angiogenesis [3].

MM cells develop complex interactions with BM-mesenchymal stromal cells (BM-MSCs) supporting tumor survival and chemoresistance [4]. Moreover, BM-MSCs appear to have reduced osteogenic potential and to stimulate proliferation/activity of osteoclasts, thus contributing to osteolysis [5]. Osteoclasts also establish a feed-forward relationship with PCs, which further supports tumor growth [6]. The role of osteoblasts and osteocytes is yet to be clarified although some authors showed MM cells to inhibit osteoblast proliferation, differentiation, and activity through different molecular pathways while promoting osteocyte apoptosis [6].

In BM microenvironment the imbalance between pro- and anti-angiogenic factors represents a key step for tumor progression [7]. To date, the underlying pathophysiology of the pro-angiogenic switch in MM is only partially understood. The release of pro-angiogenic molecules by BM cells has a non-negligible role in the loss of angiostasis in Monoclonal Gammopathy of Undetermined Significance (MGUS) pre-tumoral quiescent condition [7]. Furthermore, interactions between malignant cells and BM microenvironment have been shown to enhance angiogenesis and to promote tumor progression in a vicious circle [8]. MM progression appears to be accompanied by an increase in angiogenesis from the MGUS pre-vascular/non-active phase to the vascular phase characterizing active MM [8]; [7].

Since first identification, MSCs have been associated to bone turnover and metabolism in a number of pathological conditions [9], including MM [5]. The secretion of cytokines and stimulating factors points to MSC involvement in vascular growth [10] although MSC ability to differentiate directly into endothelial cells is still debated. *In vitro* studies showed contradictory results possibly due to the heterogeneity of bulk cultures [11]. Indeed, culture conditions can select diverse sub-populations of MSC progenitors with specific differentiation potentials, as shown by the effect of human sera on the isolation of Mesangiogenic Progenitor Cells (MPCs) [12].

MPCs have been identified in human BM under specific culture condition. Cells are round shaped with a refractive central core and fringed periphery, and they show trypsin-resistant plastic adherence [13]. MPCs express pluripotency-associated genes [14] and have been characterized as resting cells, retaining both mesengenic and angiogenic potential [15]. In particular, MPC mesengenic differentiation is a two step process. First, activation of non-canonical Wnt-5/calmodulin pathway drives cells to the P1-MSC stage. Addition of calmodulin antagonist calmidazolium chloride (CLMDZ) [16], during this step, results in the complete ablation of the mesengenic differentiation while have no effect on resting MPCs or their endothelial differentiation [17]. These data not only confirmed the activation of calmodulin during the induction of MPCs into P1-MSCs, but also correlate the sensibility to CLMDZ with this specific step of mesengenic differentiation. In fact, the second passage under mesengenic stimulation, leading P1-MSC into P2-MSC showing standard MSC morphology, phenotype and function, is not affected by CLMDZ treatment and apparently involving the canonical Wnt pathway [17]. MPCs can undergo the angiogenic fate under VEGF stimulus by two step culture, with angiogenesis prompted by MPC 3D-spheroids let sprouting in extracellular matrix protein gel. MPC angiogenic potential is lost after mesengenic induction, confirming the two fates to be mutually exclusive [15]. As stated above, the MPC endothelial differentiation is not impaired by the addition of CLMDZ, however a specific inhibitor of the MPC angiogenic fate has not been tested before. In 2016, a specific BM cell population has been identified by multicolor flow cytometry as the putative and unique MPC *in vivo* progenitor [18]. Back-gating lineage markers on CD18 *vs* CD31 scatter plots allowed identification of seven clusters associated to most of the BM mononuclear cell populations. An eighth population (Pop#8) has been also identified and characterized as CD45^dim^CD33^+^CD11b^neg^CD64^bright^CD31^bright^CD14^neg^, resembling the phenotype of immature monocyte precursors. Interestingly, sorting experiment demonstrated that this latest population is the unique BM population able to generate MPCs in culture, identifying Pop#8 as the *ex vivo* ancestor of those mesangiogenic cells [18].

From the first evidence of their mesangiogenic potential, we hypothesize a role for MPCs in BM stroma re-modeling and homeostasis. However, a definitive demonstration of the involvement of MPCs in the maintaining the bone marrow microenvironment is still lacking. Nonetheless, due to their differentiation potential, it is reasonable hypothesize that the MPC behavior could be altered during MM development and progression as a consequence of the deregulation that malignant PCs exert on BM stroma. Here we investigated possible alteration of the MPC *in vitro* properties, analyzing MPC frequency and characterizing their mesangiogenic potential in MM patients and compared to non-haematological (NH) subjects. We also tested the effect of bortezomib (BTZM), the first pretoasome inhibitor applied in the treatment of MM patients with potent anti-angiogenic activity in bone marrow [19], on MPC angiogenic fate.

## RESULTS

### PC bone marrow infiltration reduces the percentage of *Pop#8* sub-population, in MM patients.

**Figure 1:**
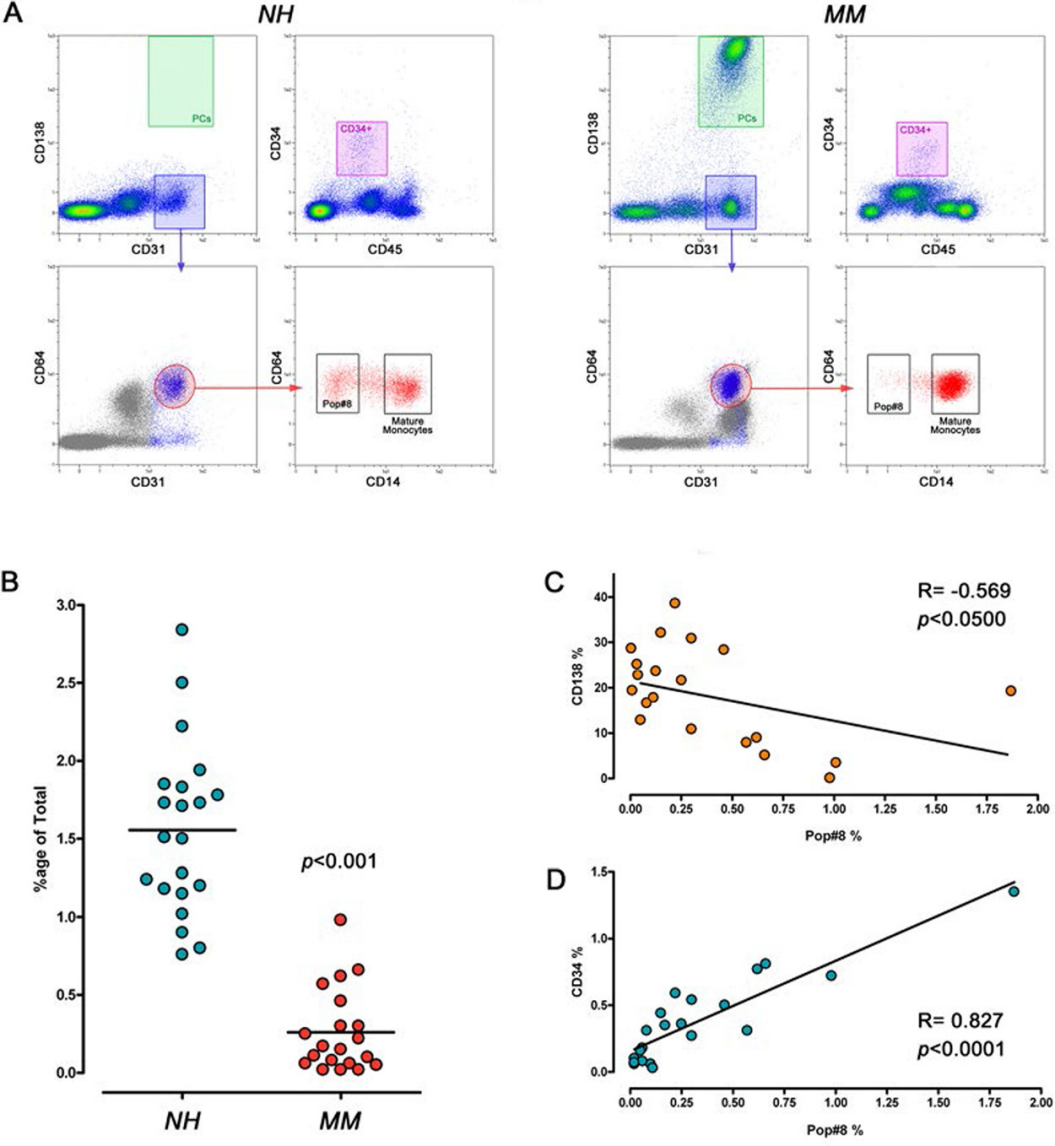
Frequency of Pop#8 sub-population in MM patients. **(A)** MPCs and haematopoietic progenitors were identified by flow cytometry as CD31^bright^CD64^bright^CD14^neg^ (black box) and CD34^+^CD45^dim^ (purple box), respectively. Malignant PCs were identified as CD138^bright^CD45^dim^ (green box). **(B)** Frequency of Pop#8 was significantly lower in MM patients as compared to NH patients. **(C)** Pop#8 frequency negatively correlated with the percentage of PCs and **(D)** was associated to reduced haematopoiesis.

The percentage of Pop#8 sub-population was significantly lower (*p*<0.001) in BM from MM patients (0.26 ± 0.06%, n=21) as compared to NH patients (1.56 ± 0.12%, n=20) (Figure 1A, 1B). Such reduced frequency could be the consequence of malignant PC infiltration in MM patients that has been shown to reach up to 70% of BM, resulting in hypoplasia of the main haematopoietic lineages. Indeed, absolute count of Pop#8 cells resulted not significantly different (52.5 ± 8.6 cells/μl, n=20 in NH *vs* 41.6 ± 10.2 cells/μl, n=21 in MM, *p*=0.424) and its percentage negatively correlated with the percentage of CD138^bright^CD45^dim/neg^ (Spearman *R* = -0.569, *p*<0.05) that ranged from 0.80% to 38.60% of total BM cells, in MM samples (Figure 1C). Moreover, positive correlation (Spearman *R* = 0.827, *p*<000.1) between Pop#8 and CD34^+^ cell frequency was detected, confirming that reduction in Pop#8 sub-population was coupled with reduced haematopoiesis (Figure 1D). Similar results were obtained for the mature monocyte population (74.3 ± 13.9 cells/μl, n=20 in NH *vs* 77.3 ± 14.7 cells/μl, n=21 in MM, *p*=0.890)

### MPCs from MM patients are forced toward the angiogenic fate, under mesengenic stimuli

**Figure 2:**
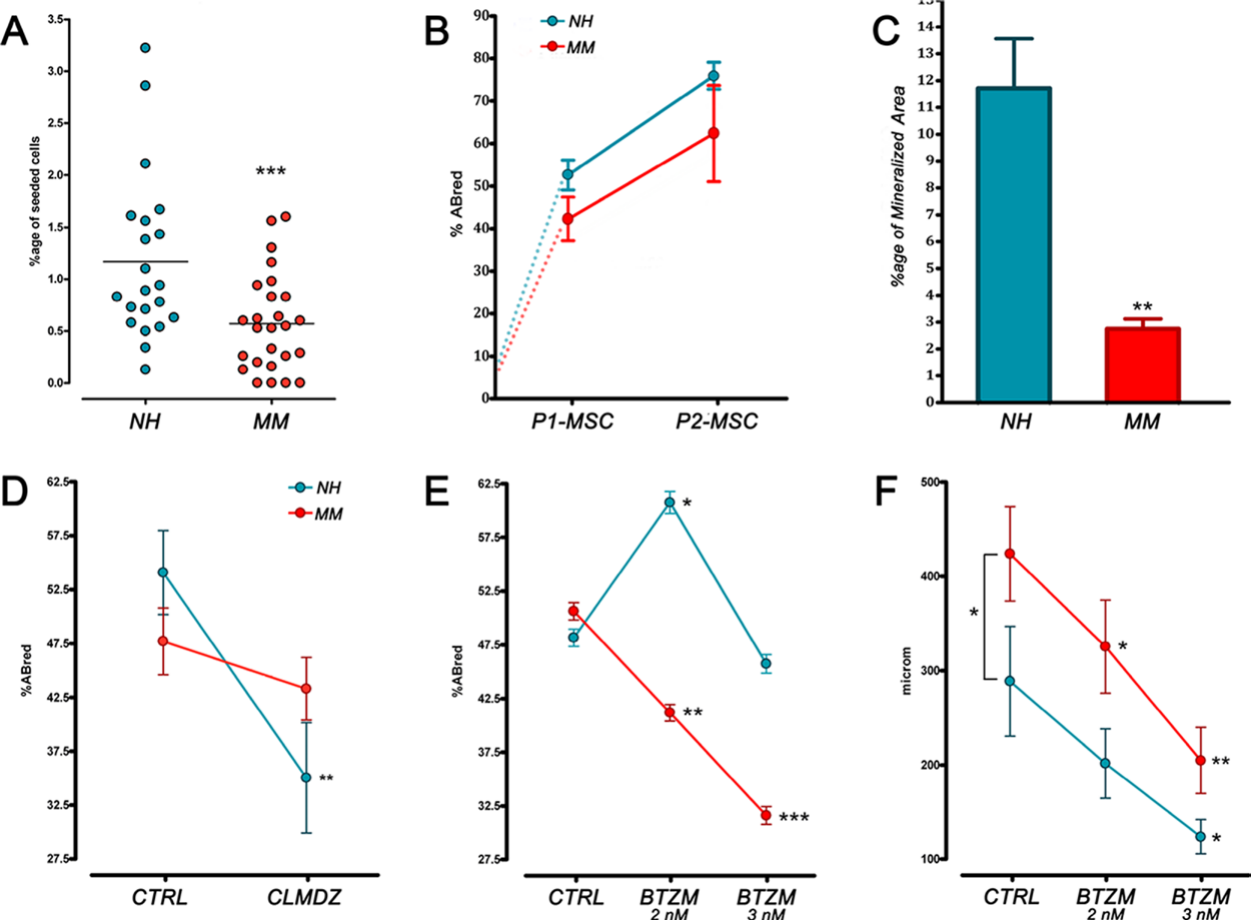
MPC frequency and differentiative potential in NH and MM patient. **(A)** MPC frequency from primary cultures was found to be significantly lower in MM samples. **(B)** Under mesengenic stimuli no difference was found between MM and NH patient-derived MPCs, neither in growth curves. **(C)** After 21 days of terminal osteogenic differentiation, a reduced mineralized area was detected in MM patients indicating defective calcium deposition. **(D, E)** BTZM inhibited MM patient-derived MPC differentiation into P1-MSC at a difference with CLMDZ. **(F)** Under angiogenic stimuli sprouting from MPC 3D spheroids resulted in longer sprouting distances in MM samples. BTZM impaired sprouting in a dose-dependent manner. (^*^ *p*<0.05, ^**^ *p*<0.01, ^***^ *p*<0.001)

After a week of culture under MPC selective conditions, three samples of the NH and two of the MM group were excluded from the study due to a low yield/purity of the recovered cell population. In the remaining samples, the mean purity of MPC cultures resulted higher than 90% both for NH (96.7 ± 1.5%, n=20) and MM samples (94.3 ± 2.6%, n=21) with undetectable CD14^bright^ population, as expected applying selective culture [20]. In accordance to the observed reduction of Pop#8 in MM patients, MPC frequency from primary cultures was found to be significantly (*p*<0.001) lower in MM samples (0.57 ± 0.09%, n=21) as compared to NH samples (1.17 ± 0.17%, n=20) (Figure 2A).

During the *in vitro* mesengenic differentiation toward P2-MSCs, no difference was found between MM and NH patients, indicating MPC mesengenic potential to apparently remain unaffected. Also, similar growth curves were recorded (Figure 2B). However, terminal osteogenic differentiation resulted impaired in P2-MSCs derived from MM patients. The significant (*p*<0.01) reduction of mineralized area in MM samples (2.88 ± 0.38%, n=21) as compared to NH (11.73 ± 1.91%, n=20) casted doubts on the genuineness of mesengenic MPC differentiation into MSC-like cells (Figure 2C). The hypothesis was confirmed by the calmodulin signalling pathway inhibition induced by CLMDZ during MPC differentiation into P1-MSCs. Addition of 0.5 μM CLMDZ resulted in about 35% inhibition of the AlamarBlue reduction in NH samples (35.05 ± 5.11%) as compared to untreated cultures (54.09 ± 3.89%) (n=12; *p*<0.01). Conversely, MPC mesengenic differentiation in MM samples was unaffected by CLMDZ treatment (43.31 ± 2.91% *vs* 47.70 ± 3.10% of untreated cultures, n=12), supporting the hypothesis that the recorded proliferation could not be associated to genuine mesengenic differentiation (Figure 2D). Treatment with 2 nM BTZM significantly (*p*<0.01) promoted MPC mesengenic differentiation in NH samples (60.63 ± 2.91% *vs* 47.93 ± 4.16% of untreated cultures, n= 16), whereas 3 nM BTZM had no effect (40.73 ± 3.44%, n=16), showing that BTZM posses a pro-osteogenic and an anti-angiogenic activity on normal MPC similarly to what reported on other bone marrow stromal cells [19], [21]. Conversely, BTZM impaired the proliferation of MPCs from MM patients during differentiation into P1-MSCs, in a dose-dependent manner (41.19 ± 2.82%, *p*<0.01, at 2 nM and 31.62 ± 2.99%, *p*<0.001, at 3 nM *vs* 50.63 ± 2.91% of untreated cultures, n=13) (Figure 2E), suggesting that the differentiation occurred could not be mesengenic but angiogenic.

MM patient-derived MPCs showed increased angiogenic potential with significantly (*p*<0.05) longer sprouting distances (442.2 ± 49.5 μm, n=16) as compared to NH patients (288.8 ± 58.3 μm, n=10). BTZM impaired sprouting angiogenesis in a dose dependent-manner, both in NH and MM patient-derived MPCs, showing similar inhibition curve slopes (Figure 2F).

**Figure 3:**
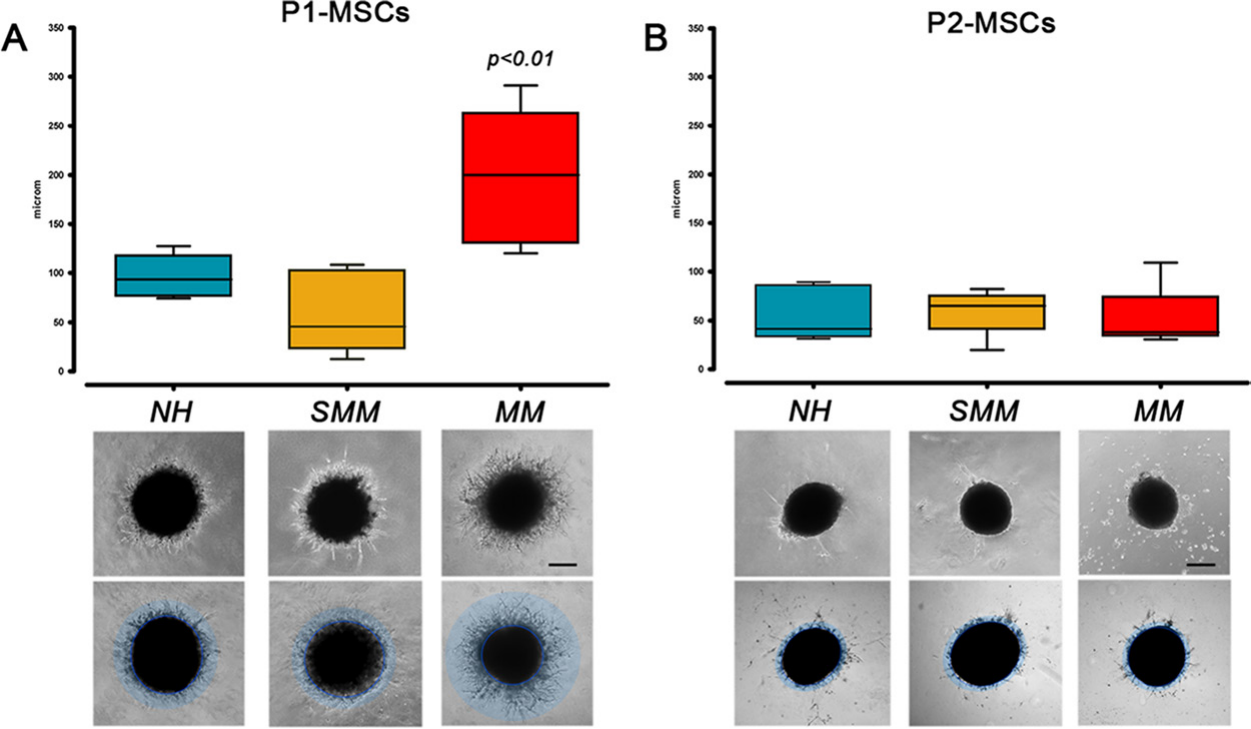
Angiogenic potential after mesengenic induction. **(A)** P1-MSCs from MM patients retained higher sprouting activity under angiogenic stimuli with respect to P1-MSCs from SMM and NH patients. **(B)** This residual angiogenic potential was lost after the final step of mesengenic differentiation to P2-MSCs. (scale bar = 200 µm)

Assessement of angiogenic potential after MPC mesengenic induction revealed significant differences between “active disease” (MM) and SMM patients (*p*<0.001) as well as between MM and NH patients (*p*<0.01). P1-MSCs from MM patients showed moderate reproducible sprouting activity with a front of invading cells at around 200 μm from the spheroid edge (197.1 ± 22.9 µm, n=10) at a difference with P1-MSCs from NH and SMM patients that revealed branching of cellular protrusions only within 100 µm from the spheroid edge (96.5 ± 22.9 µm, n=6 and 57.8 ± 14.67 µm, n=8, respectively) (Figure 3A). This data further support the idea that P1-MSCs from MM patients were not mesenchymal cells but early angiogenic cells, as suggested by the inhibition experiments with CLMDZ and BTZM. The residual angiogenic potential of MM P1-MSCs was lost after the second step of mesengenic differentiation, as shown by lack of sprouting activity in P2-MSC derived spheroids, similarly to previous results in orthopedic patients [15]. Taking together our results strongly support a forced angiogenic fate for MPCs from MM patients, during the differentiation into P1-MSCs.

### Gene expression profiling of P1-MSCs supports the hypothesis of forced MPC angiogenic fate

**Figure 4:**
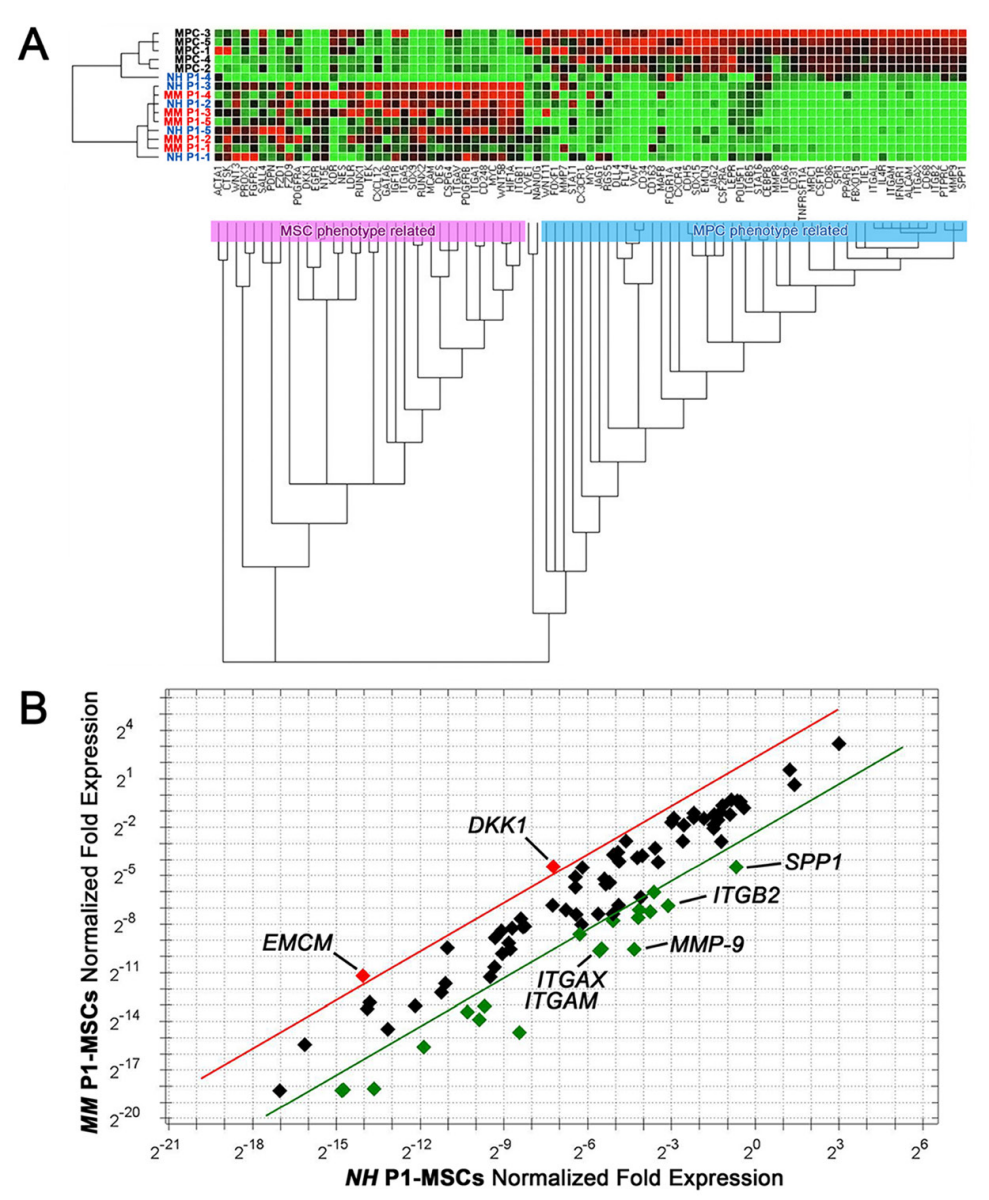
Gene expression profile of P1-MSCs from NH and MM patients. **(A)** Clustering gene expression analysis of samples from NH (blue font) and MM (red font) patients showed up-regulation of MSC-related genes (red dots) and down-regulation of MPC-related genes (green dots). **(B)** Single gene expression analysis revealed increased expression of *DKK1* and *EMCN* in MM samples (red dots). Specific MPC markers showed increased expression in NH samples (green dots). Five-fold ratio of normalized fold expression was fixed as a threshold (red and green lines).

Unsupervised gene expression clustering analysis of 86 target genes revealed two main clusters associated to the MPC (pale blue box) or MSC (pink box) phenotype (Figure 4A). After mesengenic induction, P1-MSC samples from NH and MM patients both showed increased expression of MSC-related genes and reduced expression of MPC-related genes. Single gene analysis revealed lower expression of the most specific previously described MPC markers, including osteopontin (*SPP1*), CD18 (*ITGB2*), CD11b (*ITGAM*), CD11c (*ITGAX*), and matrix metallopeptidase 9 (*MMP9*), from 0.07 to 0.03 fold, in MM samples (n=5, Figure 4B). The down-regulation of MPC-associated genes is expected during differentiation into P1-MSCs, however in MM samples silencing of these genes resulted about 2 logs more consistent respect to NH (*SPP1*: -5.81 x10^2^ *vs* -4.00 x10^1^; *ITGB2*: -1.10 x10^3^ *vs* -7.68 x10^1^; *ITGAM*: -1.50 x10^3^ *vs* -8.77 x10^1^; *ITGAX*: -1.50 x10^3^ *vs* -6.5 x10^1^; *MMP9*: -4.54 x10^3^ *vs* -1.13 x10^2^, *p*<0.05, n=5). Conversely, a seven-fold higher expression of Dickkopf WNT signaling pathway inhibitor 1 (*DKK1*, 6.96 ± 2.87, n=5, *p*<0.05) and endomucin (*EMCN*, 7.28 ± 3.59, n=5, *p*<0.05) has been detected in P1-MSCs derived from MM samples. These data suggest that, in MM patients, the unexpected angiogenic potential at P1-MSCs is not correlated to the maintaining of the MPC undifferentiated condition. Moreover, the up-regulation of *DKK1* could be suggestive for a hampered mesengenic fate, associated to the activation of canonical Wnt/β-catenin signaling.

## DISCUSSION

In recent years impairment of bone turnover and angiogenesis have been described as two hallmarks of tumor microenvironment in MM [22]; [23]. The microenvironment has a fundamental role in supporting MM, since malignant PCs survive almost exclusively in BM [3]. Indeed, novel treatment strategies target malignant tumor cells as well as their microenvironment, allowing deep responses and fuelling the debate cure *vs* long-term response [24]. In the present study, we showed altered *in vitro* response to differentiation stimuli of MPCs derived from MM patients, suggesting that these cells could play a role in: i) tumor growth, contributing to new vessels formation and ii) osteolysis, reducing the osteblastogenesis.

It is known that BM infiltration by myeloma cells reduce physiological hematopoiesis due to the invasion of BM cavity [25]. Consistently, in our study BM resident MPC precursor *Pop#8* cells [18] and classical monocytes, although were found at lower frequencies as a consequence of tumor burden, resulted unvaried in their absolute count in samples from MM patients as compared to NH patients.

As regard to MPC differentiative potential, we found apparently no significant difference in mesengenic differentiation to P2-MSCs between NH and MM patients. On the other hand, osteogenic terminal differentiation was significantly compromised in patients diagnosed with MM. A similar behavior had been related to inhibition of the non-canonical Wnt5a/Ror2 pathway during MSC differentiation to osteoblasts in MM [26]. In our experimental setting data suggest a switch, possibly induced by malignant PCs, from genuine mesengenic differentiation leading to MSC-like cells in favor of a different MPC fate. BTZM treatment inhibited MM patient-derived MPC differentiation into P1-MSC at a difference with CLMDZ, supporting the idea of MPC fate restriction notwithstanding the observed defective osteogenic terminal differentiation. We previously demonstrated Wnt5a non-canonical pathway involvement in MPC differentiation to P1-MSCs [17] and also evidenced the presence of undifferentiated MPCs within MSC bulk cultures [11]. Therefore, the osteogenic differentiation impairment described by Bolzoni *et al*. [26] would be a consequence of MSC bulk culture heterogeneity. In our scenario, CLMDZ treatment could act on MPCs rather than MM-derived MSCs re-balancing their differentiative potential toward mesengenesis. On a larger scale one could envisage the inhibition of musculoskeletal fate in MM patients to be localized at the stage of MPCs rather than MSCs.

MM patient-derived MPCs showed increased angiogenic potential as compared to NH patients. BTZM impaired sprouting angiogenesis in a dose dependent-manner, both in NH and MM MPCs. Assessement of angiogenic potential after MPC mesengenic induction revealed significant differences between “active disease” (MM) and SMM patients as well as between MM and NH patients. P1-MSCs from MM patients still showed moderate reproducible sprouting activity proving a persistent angiogenic potential. This latest was lost after the second step of mesengenic differentiation, similarly to previous results in orthopedic patients [15] except for the defective osteogenic terminal differentiation evidenced by the inefficient calcium deposition. Our set of data is indicative of progressive loss of MPC angiogenic potential without restoration of their *in vitro* osteogenic activity once abandoned the *in vivo* conditioning myelomatous BM microenvironment. The forced angiogenic fate of MPCs appears to be related to MM active stage, since sprouting activity in SMM patients was comparable to NH patients.

Gene expression analysis of a set of genes involved in MPC mesenchymal differentiation revealed overlapping profiles between NH and MM derived P1-MSCs. The only exception was represented by a consistent up-regulation of endomucin (*EMCN*), recently described as a marker for type H vessels. This sub-set of BM microvessels has been proposed to mediate local growth of vasculature and their localization at the distal end of the arterioles suggest for them to support a specialized metabolically active microenvironment, both in mice and humans [27]; [28]. Creating a privileged access to nutrients and oxygen, CD31^hi^Emcn^hi^ capillaries may represent a key factor sustaining malignant BM cells. *DKK1*, whose down-regulation has been detected during the late stage of MPC mesengenic differentiation into P2-MSCs [17], showed increased expression in MM derived P1-MSCs. Such up-regulation could prevent differentiation into genuine MSC-like cells and force angiogenic commitment.

Prior studies described BTZM effect on tumor microenvironment focusing on the induction of osteogenic terminal differentiation [21]; [29]. Our *in vitro* evidence provides a possible additional effect of BTZM treatment. Basing on the assumption that mesenchymal differentiation in MM is partly blocked at the stage of MPCs that are forced toward angiogenesis, we hypothesize that malignant PCs could modify the MPC fate in favor of tumor growth and consequently affect osteogenesis. BTZM anti-angiogenic effect would therefore be able to revert the MPC angiogenic switch restoring MPC mesengenic potential. We also believe that the unbalance in the angiogenic potential shown by MM *vs* SMM derived P1-MSCs would localize MPC forced angiogenesis *in vivo* at the switch from asymptomatic SMM to active MM. In this scenario, BTZM activity on MPCs not only would be able to restore a balanced mesangiogenesis in MM, but also in SMM could act protecting from the angiogenic switch, at the basis of the disease progression. Even if further data supporting this hypothesis are needed, our results could therefore have relevant clinical implications to improve patients’ outcome. Current recommendations continue to be SMM patient observation. New indications of therapy would contribute to prevent progression ad hopefully eradicate the disease at early stage [30]. The unaffected mesenchymal and osteoblastic differentiation we observed under low tumor burden supports the idea of treating SMM patients before substantial microenvironmental deregulation [31]. Moreover, in agreement with previous studies focused on the role of proteasome inhibitors for post-transplant maintenance [32]; [33]; [34]; [35], we believe that BTZM could establish a non-permissive tumor microenvironment by blocking the MPC angiogenic switch, thus deepening or maintaining disease remission.

In conclusion, our *in vitro* cell system appeared to be consistent with the natural history of MM, suggesting a similar MPC behavior *in vivo*. Our data allowed to show MM angiogenesis and osteolysis not be seen as independent aspects of the same disease, but rather as coupled processes responsible for tumor progression. We also evidenced the expression of a number of genes, including *ECMN* and *DKK1*, possibly involved in the deregulation of tumor microenvironment. Further studies will be required to evaluate whether targeting the MPC fate could interfere with tumor growth and restore osteogenesis, as well as hampering disease progression.

## METHODS

### Bone marrow mononuclear cell isolation

Human BM aspirates were collected by iliac crest puncture from 32 patients (16F/16M, median age 68, range 52-85) after written consent (Supplementary Table 1). The experimental group included 23 MM (7 I*SS I*, 11 *ISS II*, and 3 *ISS III*) patients (15 with bone lytic lesions) and 9 Smoldering Multiple Myeloma (SMM) patients, either newly diagnosed or free from therapies for almost two years [36]. No patients with solitary myeloma or extra medullary plasmocytoma were enrolled. BM aspirates were also collected from 23 control patients (13F/10M, median age 66, range 50-72) with non hematological disease (NH). Samples were collected during hip replacement orthopedic surgery immediately after femoral neck osteotomy. The study was performed according to the declaration of Helsinki and sample collection protocol approved by the ethical committee of the Azienda Ospedaliero-Universitaria Pisana (committee approval number: 48812/07).

BM samples were diluted 1:4 in Dulbecco’s modified phosphate-buffered saline (D-PBS; Thermo Fisher, Waltham, MA-USA) and gently layered on Ficoll-Paque^®^ PREMIUM (GE Healthcare, Uppsala, Sweden). Samples were centrifuged at 400 g for 25 min and BM mononuclear cells (BM-MNCs) harvested at the interface, washed twice in D-PBS and counted by Bürker hemocytometer.

### Flow cytometry

Freshly isolated BM-MNCs were processed for multicolor flow cytometry analysis. Cells were incubated for 30 min at 4° C with fluorochrome-conjugated antibodies: anti-CD34 VioBlue^®^, anti-CD64 FITC, anti-CD138 PE, anti-CD14 PerCP-Cy5.5, anti-CD31 PE-Cy7, anti-CD56 APC, and anti-CD45 APC-Vio770^®^ (Miltenyi Biotec, Bergisch Gladbach, Germany). After washing in MACSQuant^®^ Running Buffer (Miltenyi Biotec), cells were sorted by MACSQuant^®^ Analyzer (Miltenyi Biotec) equipped with MACSQuantify^®^ analysis software (Miltenyi Biotec). After exclusion of debris on SSC *vs* FSC plot and doublet on FSC-H *vs* FSC-A, events were plotted on CD138 *vs* CD31 density plot. Two different gates were identified to select CD138^bright^CD31^+^ and CD138^neg^CD31^bright^. CD138^bright^CD31^+^ were then plotted on CD45 *vs* CD138 and CD138^bright^CD31^+^CD45^dim/neg^ events recorded as PCs. As previously reported, two further sub-gates of the CD138^neg^CD31^bright^ population were defined for accurate quantification of *Pop#8* sub-population as CD31^bright^CD64^bright^CD14^neg^ [18] and mature monocytes as CD31^bright^CD64^bright^CD14^bright^, CD45^dim^CD34^+^ cells were also quantified (Figure 1A). Frequencies of all those cell sub-populations were calculated on singlet cellular events obtained excluding debris on SSC *vs* FSC and doublets on FSC-A *vs* FSC-H plots. Non parametric Wilcoxon’s test for matched pairs and Spearman correlation test were performed by GraphPad Prism^®^ software (GraphPad Software, San Diego, USA-CA). Absolute count of cells per μl was also recorded and the mean values were compared applying *t*-test.

### BM-MNC culture under MPC selective conditions

MPCs were isolated from BM-MNC cultures as previously described [20]. Briefly, cells were seeded from 6x10^5^/cm^2^ to 8x10^5^/cm^2^ on no gas-treated hydrophobic plastic flasks (GreinerBio-One, Kremsmünster, Austria) and cultured in Dulbecco’s modified Eagle’s medium (DMEM, Thermo Fisher) supplemented with 10% pooled human AB-type serum (PhABS, SeraLab, West Sussex, UK). Serum lots had been screened to provide optimal MPC isolation. Media were changed every 48h, and cultures maintained at 37°C in 5% CO_2_, for 6-7 days. Cells were then detached by TrypLE Select^®^ (Thermo Fisher) digestion and counted in Bürker hemocytometer. Flow cytometry was also applied to validate the MPC preparations. Around 100’000 cells from freshly detached MPC were stained with anti-CD45 APC-Vio770^®^, anti-CD31 PE-Cy7, anti-CD18 FITC, anti-CD73 PE and anti-CD90 APC (Miltenyi Biotec). Cultures with MPC population, defined as CD31^+^CD18^+^CD45^low^CD73^neg^CD90^neg^, lower than 90% were excluded from the study.

MPC frequency was calculated as ratio between MPCs obtained at the end of culture and total number of seeded cells. Statistical significance was assessed by Wilcoxon’s test.

### Assessment of MPC differentiative potential

MPC mesengenic, osteogenic, and angiogenic differentiation pathways were induced using previously described protocols [20].

#### Mesengenesis

Freshly detached MPCs obtained as above were seeded at 2x10^4^ cells/cm^2^ in TC-treated T25 flasks and let to adhere in DMEM/10% PhABS. After 24 h medium was replaced with StemMACS^™^ MSC Expansion Media XF (Miltenyi Biotec) and cultures grown to confluence to obtain passage one MSCs (P1-MSCs). Cells were then detached with TrypLE Select^®^ and sub-cultured 1:2 to confluence to obtain passage two MSCs (P2-MSCs). In parallel, 6-well plates were set up in duplicate to draw corresponding growth curves. AlamarBlue reduction assay was performed at day 7 and day 14 adding 10% PrestoBlue^®^ Cell viability assay (Thermo Fisher) to culture medium. After 8 h incubation, 100 μl of culture medium were harvested and absorbance was measured at 570 and 600 nm by Benchmark Plus microplate spectrophotometer (BioRad, Hercules, USA-CA). Percentage of reduced AlamarBlue was calculated according to manufacturer and two-tailed unpaired *t*-test was performed. Growth curves were also acquired after cell treatment with 0.5 μM calmidazolium chloride (CLMDZ) (Sigma Aldrich, St. Louise, USA-MO) or 2 and 3 nM bortezomib (BTZM) (Selleckchem, Houston, USA-TX).

#### Osteogenesis

P2-MSCs were detached, re-plated at 20.000 cells/cm^2^ in 24-well TC-treated plates, and grown to confluence in MSC expansion medium. To induce osteogenesis, medium was replaced by StemMACS™ OsteoDiff Medium (Miltenyi Biotec) and cultures maintained for 21 days (medium changed weekly). Control plates were set up in MSC expansion medium. Calcium deposits were revealed by OsteoImage™ Mineralization assay kit (Lonza, Basel, Switzerland) according to manufacturer. Pictures were taken using an inverted fluorescence DM IRB Leica microscope (Leica) equipped with LAS image acquisition software (Leica). Image analysis was performed by QWin image analysis software (Leica) to quantify the percentage of fluorescent (mineralized) areas. Two-tailed unpaired *t*-test was performed.

#### Sprouting angiogenesis

3D spheroids (6 to 8) were obtained by hanging drop method applying 1.5x10^5^ cells/drop, as previously described [15]. After 24 h, spheroids were gently laid on Geltrex^®^ (Thermo Fisher) thick gel and cultured for one week in EGM-2 medium (Lonza, Basel, Switzerland) with a single medium change. Sprouting angiogenesis was assayed on MPC derived spheroids, both in the presence (2 and 3 nM) or absence of BTZM, as well on P1- and P2-MSCs derived spheroids in the absence of BTZM. All samples were run in duplicate. Phase contrast pictures were taken at 100X magnification and sprouting distances quantified by QWin image analysis software (Leica) measuring the distance between the last invading cell and the spheroid edge. Measures were taken in blind by three separate researchers. Statistical analysis was performed by two-tailed unpaired *t*-test.

### Gene expression analysis of P1-MSCs

Gene expression analysis was performed in P1-MSCs, using NH-derived MPCs as undifferentiated control. Custom 96-well PrimePCR™ Plates (BioRad, Hercules, USA-CA) containing primer sets for 86 target genes, 5 reference genes (Supplementary Table 2), and 5 internal controls were used for the gene expression profile assay. Total RNAs were purified from freshly detached cells by Direct-zol RNA MicroPrep Kit (Zymo Research, Irvine, USA-CA) and quantified with Qubit 4 Fluorometer (Thermo Scientific) by Qubit RNA HS Assay Kit (Termo Scientific). cDNAs were synthesized from 1 μg total RNA using iScript gDNA Clear cDNA Synthesis Kit, according to manufacturer. qPCR was carried out by SsoAdvanced Unversal SybrGreen Supermix (BioRad) on iQ5 Real time PCR Detection System (BioRad), according to PrimePCR Array™ manufacturer. Fold change ^ΔΔ^C_t_ method calculations and statistical analysis were carried out using PrimePCR™ Analysis software (BioRad). C_t_ values over 35 were considered as “no expression”. Following best housekeeping gene test, *B2M* and *GAPDH* were selected for normalization.

## SUPPLEMENTARY MATERIALS AND FIGURES







## Abbreviations

BM: bone marrow
BM-MNCs: bone marrow mononuclear cells
BTZM: bortezomib
CLMDZ: calmidazolium chloride
DSS: Durie-Salmon stage
ISS: international scoring system
MGUS: monoclonal gammopathy of undetermined significance
MM: multiple myeloma
MPC: mesangiogenic progenitor cells
NH: non-hematological
MSCs: mesenchymal stromal cells
PCs: plasma cells
SMM: smoldering multiple myeloma
VEGF: vascular-endothelial growth factor.
