# Supplementary material for: Mesangiogenic progenitor cells are forced toward the angiogenic fate, in multiple myeloma
Source: Oncotarget. 2019 Nov 26;10(63):6781–90. doi: 10.18632/oncotarget.27285 (PMC6887577; doi:10.18632/oncotarget.27285)
Supplement: Supplementary file 1 [file oncotarget-10-6781-s001.pdf]

Mesangiogenic progenitor cells are forced toward the angiogenic fate, in multiple myeloma

SUPPLEMENTARY MATERIALS

Supplementary Table 1: Patients enrolled in the study.

Abbreviations: ID Code (identification code); PC(%) (percentage of infiltrating PCs in BM); DSS (Durie-Salmon Stage); ISS (International Scoring System)

Supplementary Table 2: Primer sets applied for gene expression profiling.
