# Supplementary material for: Mesangiogenic progenitor cells are forced toward the angiogenic fate, in multiple myeloma
Source: Oncotarget. 2019 Nov 26;10(63):6781–90. doi: 10.18632/oncotarget.27285 (PMC6887577; doi:10.18632/oncotarget.27285)
Supplement: Supplementary file 3 [file oncotarget-10-6781-s003.docx]

| **Well** | **Gene Name** | | | **Gene Symbol** | **Entrez Gene ID** | | **Unique Assay Id** | | **Amplicon Length** | |
| --- | --- | --- | --- | --- | --- | --- | --- | --- | --- | --- |
| A1 | actin, alpha 1, skeletal muscle | | | ACTA1 | 58 | | qHsaCED0045151 | | | 60 |
| A2 | cadherin 5, type 2 (vascular endothelium) | | | CDH5 | 1003 | | qHsaCID0016288 | | | 112 |
| A3 | chemokine (C-X-C motif) receptor 4 | | | CXCR4 | 7852 | | qHsaCED0002020 | | | 142 |
| A4 | fibroblast growth factor receptor 2 | | | FGFR2 | 2263 | | qHsaCID0021928 | | | 124 |
| A5 | insulin-like growth factor 1 receptor | | | IGF1R | 3480 | | qHsaCID0015667 | | | 81 |
| A6 | integrin, alpha V (vitronectin receptor, alpha polypeptide, antigen CD51) | | | ITGAV | 3685 | | qHsaCID0006233 | | | 130 |
| A7 | low density lipoprotein receptor | | | LDLR | 3949 | | qHsaCID0015114 | | | 121 |
| A8 | mannose receptor, C type 1 | | | MRC1 | 4360 | | qHsaCID0037074 | | | 77 |
| A9 | podoplanin | | | PDPN | 10630 | | qHsaCID0009013 | | | 134 |
| A10 | sal-like 4 (Drosophila) | | | SALL4 | 57167 | | qHsaCID0014611 | | | 157 |
| A11 | tyrosine kinase with immunoglobulin-like and EGF-like domains 1 | | | TIE1 | 7075 | | qHsaCID0006540 | | | 75 |
| A12 | glyceraldehyde-3-phosphate dehydrogenase | | | GAPDH | 2597 | | qHsaCED0038674 | | | 117 |
| B1 | activated leukocyte cell adhesion molecule | | | ALCAM | 214 | | qHsaCID0037887 | | | 173 |
| B2 | CCAAT/enhancer binding protein (C/EBP), beta | | | CEBPB | 1051 | | qHsaCED0019041 | | | 117 |
| B3 | desmin | | | DES | 1674 | | qHsaCID0011768 | | | 116 |
| B4 | fms-related tyrosine kinase 4 | | | FLT4 | 2324 | | qHsaCID0020886 | | | 150 |
| B5 | insulin-like growth factor 2 receptor | | | IGF2R | 3482 | | qHsaCID0018295 | | | 112 |
| B6 | integrin, alpha X (complement component 3 receptor 4 subunit) | | | ITGAX | 3687 | | qHsaCID0006223 | | | 104 |
| B7 | leptin receptor | | | LEPR | 3953 | | qHsaCID0018270 | | | 131 |
| B8 | v-myb myeloblastosis viral oncogene homolog (avian) | | | MYB | 4602 | | qHsaCED0043781 | | | 105 |
| B9 | POU class 5 homeobox 1 | | | POU5F1 | 5460 | | qHsaCED0038334 | | | 100 |
| B10 | SRY (sex determining region Y)-box 15 | | | SOX15 | 6665 | | qHsaCED0044255 | | | 78 |
| B11 | tumor necrosis factor receptor superfamily, member 11a, NFKB activator | | | TNFRSF11A | 8792 | | qHsaCID0006213 | | | 99 |
| B12 | hypoxanthine phosphoribosyltransferase 1 | | | HPRT1 | 3251 | | qHsaCID0016375 | | | 90 |
| C1 | platelet and endothelial cell adhesion molecule 1 | | | PECAM1 | 5175 | | * | | | 231 |
| C2 | colony stimulating factor 1 receptor | | | CSF1R | 1436 | | qHsaCID0010604 | | | 150 |
| C3 | dickkopf homolog 1 (Xenopus laevis) | | | DKK1 | 22943 | | qHsaCED0002060 | | | 118 |
| C4 | forkhead box F1 | | | FOXF1 | 2294 | | qHsaCED0002932 | | | 133 |
| C5 | interleukin 4 receptor | | | IL4R | 3566 | | qHsaCID0008648 | | | 143 |
| C6 | integrin, beta 1 (fibronectin receptor, beta polypeptide, antigen CD29) | | | ITGB1 | 3688 | | qHsaCED0005248 | | | 104 |
| C7 | lymphatic vessel endothelial hyaluronan receptor 1 | | | LYVE1 | 10894 | | qHsaCID0010430 | | | 126 |
| C8 | v-myc myelocytomatosis viral oncogene homolog (avian) | | | MYC | 4609 | | qHsaCID0012921 | | | 103 |
| C9 | peroxisome proliferator-activated receptor gamma | | | PPARG | 5468 | | qHsaCID0011718 | | | 117 |
| C10 | SRY (sex determining region Y)-box 9 | | | SOX9 | 6662 | | qHsaCED0021217 | | | 77 |
| C11 | von Willebrand factor | | | VWF | 7450 | | qHsaCED0033955 | | | 113 |
| C12 | ribosomal protein L13 | | | RPL13 | 6137 | | qHsaCED0056592 | | | 114 |
| D1 | CD163 molecule | | | CD163 | 9332 | | qHsaCID0012333 | | | 150 |
| D2 | colony stimulating factor 2 receptor, alpha, low-affinity (granulocyte-macrophage) | | | CSF2RA | 1438 | | qHsaCID0013436 | | | 92 |
| D3 | delta-like 4 (Drosophila) | | | DLL4 | 54567 | | qHsaCID0008450 | | | 84 |
| D4 | frizzled homolog 1 (Drosophila) | | | FZD1 | 8321 | | qHsaCED0018783 | | | 116 |
| D5 | integrin, alpha 1 | | | ITGA1 | 3672 | | qHsaCID0017712 | | | 144 |
| D6 | integrin, beta 2 (complement component 3 receptor 3 and 4 subunit) | | | ITGB2 | 3689 | | qHsaCED0003958 | | | 63 |
| D7 | v-maf musculoaponeurotic fibrosarcoma oncogene homolog B (avian) | | | MAFB | 9935 | | qHsaCED0002199 | | | 115 |
| D8 | Nanog homeobox | | | NANOG | 79923 | | qHsaCED0043394 | | | 158 |
| D9 | prospero homeobox 1 | | | PROX1 | 5629 | | qHsaCID0018340 | | | 86 |
| D10 | spleen focus forming virus (SFFV) proviral integration oncogene spi1 | | | SPI1 | 6688 | | qHsaCID0022097 | | | 96 |
| D11 | wingless-type MMTV integration site family, member 11 | | | WNT11 | 7481 | | qHsaCID0011927 | | | 88 |
| D12 | PrimePCR DNA Contamination Control Assay | | | gDNA |  | | qHsaCtlD0001004 | | |  |
| E1 | CD248 molecule, endosialin | | | CD248 | 57124 | | qHsaCED0018682 | | | 121 |
| E2 | chondroitin sulfate proteoglycan 4 | | | CSPG4 | 1464 | | qHsaCID0005989 | | | 148 |
| E3 | epidermal growth factor receptor | | | EGFR | 1956 | | qHsaCID0007564 | | | 97 |
| E4 | frizzled homolog 9 (Drosophila) | | | FZD9 | 8326 | | qHsaCED0019089 | | | 69 |
| E5 | integrin, alpha 5 (fibronectin receptor, alpha polypeptide) | | | ITGA5 | 3678 | | qHsaCID0021495 | | | 140 |
| E6 | integrin, beta 5 | | | ITGB5 | 3693 | | qHsaCID0007523 | | | 76 |
| E7 | melanoma cell adhesion molecule | | | MCAM | 4162 | | qHsaCID0020233 | | | 146 |
| E8 | nestin | | | NES | 10763 | | qHsaCED0001303 | | | 87 |
| E9 | protein tyrosine phosphatase, receptor type, C | | | PTPRC | 5788 | | qHsaCED0038908 | | | 69 |
| E10 | secreted phosphoprotein 1 | | | SPP1 | 6696 | | qHsaCID0012060 | | | 99 |
| E11 | wingless-type MMTV integration site family, member 3 | | | WNT3 | 7473 | | qHsaCID0015193 | | | 103 |
| E12 | PrimePCR Positive Control Assay | | | PCR |  | | qHsaCtlD0001003 | | |  |
| F1 | CD34 molecule | | | CD34 | 947 | | qHsaCID0007456 | | | 99 |
| F2 | cathepsin K | | | CTSK | 1513 | | qHsaCID0016934 | | | 147 |
| F3 | endomucin | | | EMCN | 51705 | | qHsaCED0046161 | | | 91 |
| F4 | GATA binding protein 6 | | | GATA6 | 2627 | | qHsaCED0045017 | | | 81 |
| F5 | integrin, alpha 6 | | | ITGA6 | 3655 | | qHsaCID0007188 | | | 130 |
| F6 | jagged 1 | | | JAG1 | 182 | | qHsaCID0006831 | | | 99 |
| F7 | matrix metallopeptidase 7 (matrilysin, uterine) | | | MMP7 | 4316 | | qHsaCID0011537 | | | 138 |
| F8 | 5'-nucleotidase, ecto (CD73) | | | NT5E | 4907 | | qHsaCID0036556 | | | 117 |
| F9 | regulator of G-protein signaling 5 | | | RGS5 | 8490 | | qHsaCED0045107 | | | 67 |
| F10 | signal transducer and activator of transcription 1, 91kDa | | | STAT1 | 6772 | | qHsaCID0007580 | | | 92 |
| F11 | wingless-type MMTV integration site family, member 5B | | | WNT5B | 81029 | | qHsaCID0038673 | | | 69 |
| F12 | PrimePCR RNA Quality Assay | | | RQ1 |  | | qHsaCtlD0001002 | | |  |
| G1 | CD68 molecule | | | CD68 | 968 | | qHsaCED0007025 | | | 82 |
| G2 | chemokine (C-X3-C motif) receptor 1 | | | CX3CR1 | 1524 | | qHsaCED0046543 | | | 68 |
| G3 | F-box protein 15 | | | FBXO15 | 201456 | | qHsaCID0021364 | | | 144 |
| G4 | hypoxia inducible factor 1, alpha subunit (basic helix-loop-helix transcription factor) | | | HIF1A | 3091 | | qHsaCID0014755 | | | 72 |
| G5 | integrin, alpha L (antigen CD11A, lymphocyte function-associated antigen 1) | | | ITGAL | 3683 | | qHsaCID0015523 | | | 146 |
| G6 | jagged 2 | | | JAG2 | 3714 | | qHsaCED0003193 | | | 127 |
| G7 | matrix metallopeptidase 8 (neutrophil collagenase) | | | MMP8 | 4317 | | qHsaCID0023232 | | | 135 |
| G8 | platelet-derived growth factor receptor, alpha polypeptide | | | PDGFRA | 5156 | | qHsaCID0007202 | | | 103 |
| G9 | runt-related transcription factor 1 | | | RUNX1 | 861 | | qHsaCID0037818 | | | 74 |
| G10 | signal transducer and activator of transcription 6, interleukin-4 induced | | | STAT6 | 6778 | | qHsaCED0056844 | | | 120 |
| G11 | actin, beta | | | ACTB | 60 | | qHsaCED0036269 | | | 62 |
| G12 | PrimePCR RNA Quality Assay | | | RQ2 |  | | qHsaCtlD0001002 | | |  |
| H1 | CD86 molecule | | | CD86 | 942 | | qHsaCID0021122 | | | 97 |
| H2 | chemokine (C-X-C motif) ligand 12 | | | CXCL12 | 6387 | | qHsaCID0012398 | | | 94 |
| H3 | Fc fragment of IgG, high affinity Ia, receptor (CD64) | | | FCGR1A | 2209 | | qHsaCED0034082 | | | 144 |
| H4 | interferon gamma receptor 1 | | | IFNGR1 | 3459 | | qHsaCID0013339 | | | 84 |
| H5 | integrin, alpha M (complement component 3 receptor 3 subunit) | | | ITGAM | 3684 | | qHsaCID0006023 | | | 146 |
| H6 | kinase insert domain receptor (a type III receptor tyrosine kinase) | | | KDR | 3791 | | qHsaCID0006310 | | | 140 |
| H7 | matrix metallopeptidase 9 (gelatinase B, 92kDa- gelatinase, type IV collagenase) | | | MMP9 | 4318 | | qHsaCID0011597 | | | 82 |
| H8 | platelet-derived growth factor receptor, beta polypeptide | | | PDGFRB | 5159 | | qHsaCID0013272 | | | 117 |
| H9 | runt-related transcription factor 2 | | | RUNX2 | 860 | | qHsaCED0044067 | | | 83 |
| H10 | TEK tyrosine kinase, endothelial | | | TEK | 7010 | | qHsaCID0015119 | | | 101 |
| H11 | beta-2-microglobulin | | | B2M | 567 | | qHsaCID0015347 | | | 123 |
| H12 | PrimePCR Reverse Transcription Control Assay | | | RT |  | qHsaCtlD0001001 | | | |  |
|  |  | | |  |  |  | | | |  |
|  |  | | |  |  |  | | | |  |
|  | (*) Primer pair has been specifically designed | Forward seq. | GAACCTGTCCTGCTCCATC | | | | |  | |  |
|  |  | Reverse seq. | TCAAACTGGGCATCATAAGAAAT | | | | |  | |  |
